# Kullback Leibler divergence in complete bacterial and phage genomes

**DOI:** 10.7717/peerj.4026

**Published:** 2017-11-30

**Authors:** Sajia Akhter, Ramy K. Aziz, Mona T. Kashef, Eslam S. Ibrahim, Barbara Bailey, Robert A. Edwards

**Affiliations:** 1 Computational Science Research Center, San Diego State University, San Diego, CA, USA; 2 Department of Microbiology and Immunology, Faculty of Pharmacy, Cairo University, Cairo, Egypt; 3 Department of Computer Science, San Diego State University, San Diego, CA, United States of America; 4 Department of Mathematics & Statistics, San Diego State University, San Diego, CA, USA; 5 Department of Biology, San Diego State University, San Diego, CA, USA

**Keywords:** Information theory, Metagenomics, Genomics, Genometrics

## Abstract

The amino acid content of the proteins encoded by a genome may predict the coding potential of that genome and may reflect lifestyle restrictions of the organism. Here, we calculated the Kullback–Leibler divergence from the mean amino acid content as a metric to compare the amino acid composition for a large set of bacterial and phage genome sequences. Using these data, we demonstrate that (i) there is a significant difference between amino acid utilization in different phylogenetic groups of bacteria and phages; (ii) many of the bacteria with the most skewed amino acid utilization profiles, or the bacteria that host phages with the most skewed profiles, are endosymbionts or parasites; (iii) the skews in the distribution are not restricted to certain metabolic processes but are common across all bacterial genomic subsystems; (iv) amino acid utilization profiles strongly correlate with GC content in bacterial genomes but very weakly correlate with the G+C percent in phage genomes. These findings might be exploited to distinguish coding from non-coding sequences in large data sets, such as metagenomic sequence libraries, to help in prioritizing subsequent analyses.

## Introduction

The central dogma of molecular biology describes the irreversible flow of information in biological systems from nucleic acids to amino acids, whose combinations make up the main cellular components: proteins. In principle, such flow of information is no different from other data storage and communication systems, and can thus be studied by the information theory (Shannon, 1948). Indeed, the information theory has been often applied to studying different aspects of prokaryotic and eukaryotic genomes, including, for example, genome composition (Grigoriev, 1999; Omer, Harlow & Gogarten, 2017; Roten et al., 2002), architecture (Dandekar et al., 1998; Koonin, 2009; Ochman & Davalos, 2006), coding potential (Gerdol et al., 2015; Zeeberg, 2002), order and entropy (Bohlin et al., 2012; Vinga, 2014), symmetry (Kong et al., 2009; Poptsova et al., 2009), and even the interaction among genetic variants in comparative analysis.

Previous studies used Shannon’s index (Shannon, 1948) to classify informative DNA sequences (Akhter et al., 2013; Chang et al., 2004; Chang et al., 2005; Chen et al., 2005) and find prophages in bacterial genomes (Akhter, Aziz & Edwards, 2012). Shannon’s index is now increasingly being used as a bioinformatics tool to solve problems related to either network or genome context, e.g., comparative genomics, resolution-free metrics, motif classification, and sequence-independent correlations (De Domenico & Biamonte, 2016; Vinga, 2014). Recently, a genome complexity metric was proposed, the biobit, which balances a genome’s entropic and anti-entropic components (Bonnici & Manca, 2016). Additionally, von Neumann entropy, which originated from Shannon’s classical information theory, is used as a divergence parameter that could be implemented from spectral data to human microbiome networking (De Domenico & Biamonte, 2016).

In an attempt to prioritize analysis efforts for high-throughput sequencing data, notably metagenomic data sets, we calculated the Shannon index of a representative sample of bacterial and phage genomes, and showed that the information content of the nucleotide sequence within a genome largely depends on the genome’s size and its GC content. Subsequently, we were able to predict which sequences within a metagenomic library are more likely to match sequences already deposited in public databases (Akhter et al., 2013). In the current study, we continue to explore the usefulness of the information theory by expanding our analysis to the coding potential of a genome, focusing on amino acids rather than nucleotide content. To this end, we used the Kullback–Leibler divergence (KLD) value (Kullback & Leibler, 1951) to examine biases in the amino acid composition of the potentially translated gene products (predicted proteins) encoded by a genome.

Kullback & Leibler (1951) generalized Shannon’s approach to support statistical comparisons between populations. The KLD value measures the deviation of one distribution from another distribution. Here, we hypothesized that KLD might be a good measure of the diversity of an encoded proteome. We demonstrate that KLD correlates well with an organism’s phylogeny and amino acid utilization profile, in addition to correlating with the GC content of bacterial genomes.

## Methods

### Retrieval of sequence data

All genomic data, including gene annotations and functional classification, were obtained from the public SEED database (http://pubseed.theseed.org (Aziz et al., 2012; Overbeek, Disz & Stevens, 2004)). Complete phage genome sequences were obtained from the Phantome database (http://phantome.org).

### Calculation of Kullback–Leibler divergence

Kullback–Leibler divergence (KLD) was initially calculated for 372 whole bacterial genomes and 835 complete phage genomes according to the following equation.

\begin{eqnarray*}{D}_{KL}(P\parallel Q)=\sum _{i}P(i)\log \nolimits P(i)/Q(i). \end{eqnarray*}

As used here, *P*_*i*_ is the frequency of the *i* th amino acid in a given genome *X*, and *Q*_*i*_ is the average frequency of this amino acid calculated from all complete genomes, i.e., all bacterial genomes were used for calculating *Q*_*i*_ when the given genome *X* is a bacterial genome, all phage genomes were used for calculating *Q*_*i*_ when *X* is a phage.

The same strategy was followed for the calculation of KLD for a specific subsystem (Overbeek et al., 2005); the subsystem analysis was conducted on subsystems covering 446 bacterial genomes. These were all bacterial genomes available at the time of the analysis, with reliable subsystems coverage and a minimal set of informative metadata, in addition to being known for hosting analyzed phages.

KLD for each phylogenetic class was calculated by the following equation, where n is the number of genomes in each class.

\begin{eqnarray*}1/n\sum _{j=1}^{n}\sum _{i}P(i\text{_}j)log \frac{P(i\text{_}j)}{Q(i\text{_}j)} . \end{eqnarray*}

## Results

### Kullback–Leibler divergence in bacterial and phage genomes

**Figure 1:**
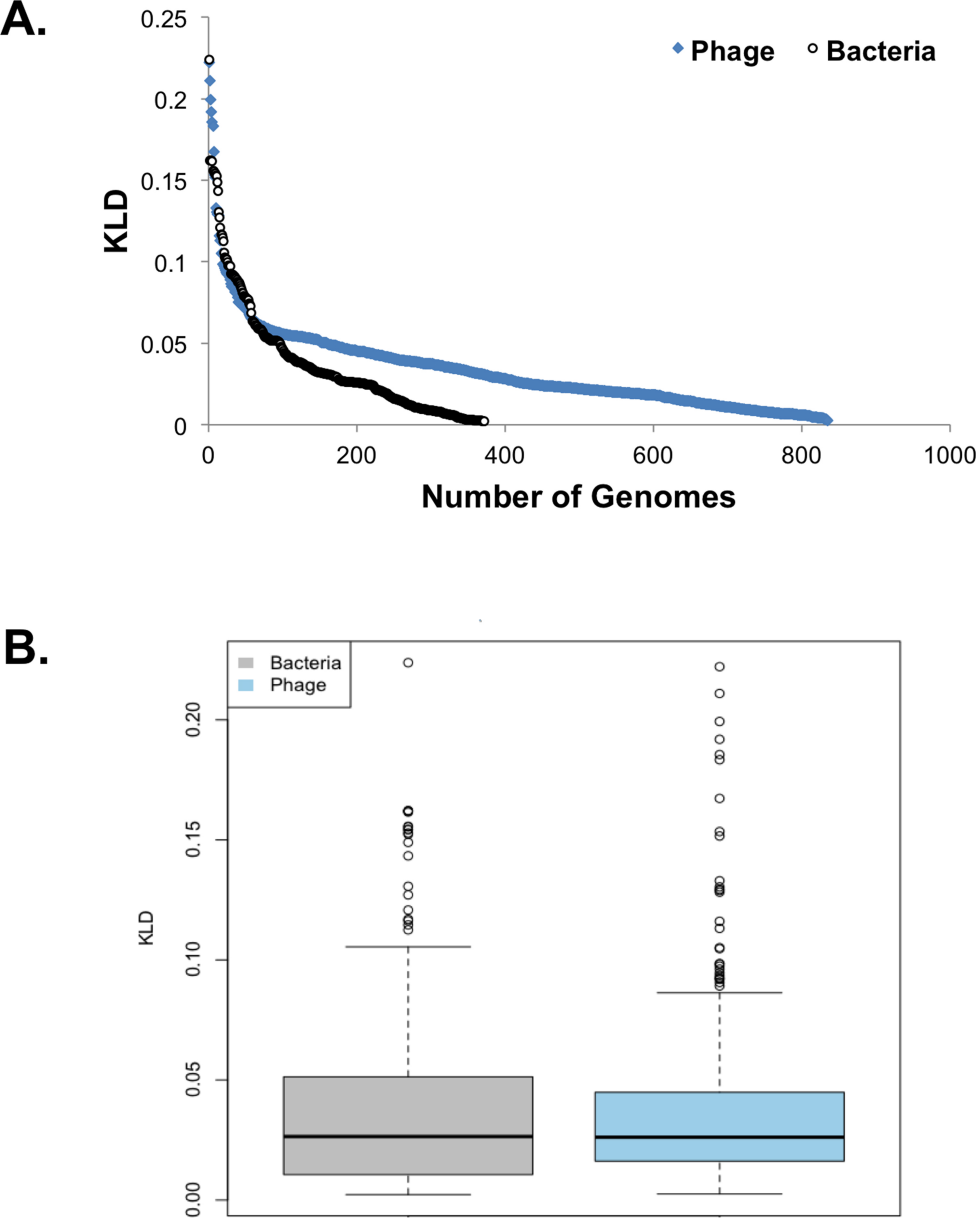
Trends in amino acid composition divergence. (A) The 372 complete bacterial genomes (black) and 835 complete phage genomes (blue) analyzed are ranked according to their composition. (B) Box plots showing the amino acid composition divergence for bacteria (gray) and phages (blue).

KLD was calculated for all predicted proteins encoded by 372 bacterial genomes and 835 phage genomes. The skew in the KLD distribution, for all genomes combined, ranged from 0.002 to 0.22 (Fig. 1). We found that both the most skewed bacterial genome, *Wigglesworthia glossinidia* (KLD = 0.224), and the most skewed phage genome, *Spiroplasma kunkelii virus SkV1_CR2-3x* (KLD = 0.222), had a low GC content of ∼22%. We also found that phage genomes have a slight tendency towards lower KLD values than bacterial genomes (Table 1). This finding suggests that bacteria might have more biased amino acid utilization than phages.

The phylogeny and lifestyle of the ten bacterial species and ten phages with the most skewed amino acid composition (as measured by their KLD values) are shown in Tables 2 and 3, respectively. Consistently, a number of bacterial species whose genomes have the most skewed amino acid compositions are parasites, and some of them are obligate intracellular parasites—with a limited ecological niche range and restricted lifestyle (e.g., *Wigglesworthia glossinidia*, an endosymbiont of the tsetse fly, Table 2). Likewise, the bacterial species that are hosts for the phage genomes with the most skewed amino acid compositions are enriched in intracellular parasites (e.g., *Spiroplasma kunkelii*, a parasite that causes Corn Stunt Disease, Table 3).

There are several possible explanations for these deviations in KLD. For example, the variation could be phylogenetically biased or determined by the lifestyle of the organism. Alternatively, a physical parameter such as the DNA composition, osmotic, or thermodynamic stability might control the variation in production of amino acids and composition of the proteins. The availability of amino acids, their precursors, or enzymatic limits on the interconversion of amino acids may also affect these skews.

**Figure 2:**
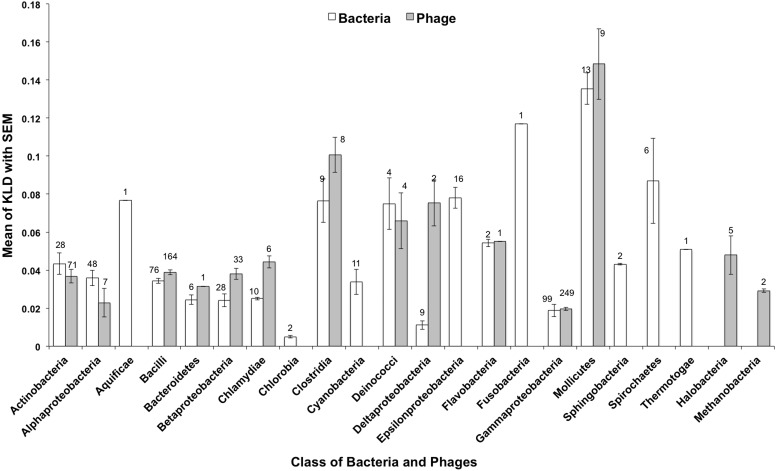
Amino acid divergence varies for each phylogenetic taxon of bacteria and phage bacterial hosts. The divergence of amino acid composition for each phyogenetic group from the mean of all bacteria and phages is shown. Error bars represent the standard error of the mean. The numbers represent the number of genomes considered for each class.

To investigate the impact of phylogeny on KLD deviations, we calculated the mean KLD for each phylogenetic group and compared it to the mean KLD of all proteins. The variation in amino acid composition provides a signature profile for each phylogenetic group (for both phage and bacterial genomes, Fig. 2), which might be predictive for metagenomic sequences.

**Table 1:** Percentage of phage and bacterial genomes in different range of KLD value.

KLD categories	Bacteria (total 372 genomes)	Phage (total 835 genomes)
KLD > 0.1	6.7% (25 genomes)	2% (18 genomes)
KLD > 0.05	25.8% (96 genomes)	19% (160 genomes)
KLD > 0.025	56% (210 genomes)	52% (435 genomes)

**Table 2:** The most skewed bacterial genomes.

Genus and Species	KLD of amino acid composition from the mean	%GC	Comments
*Wigglesworthia glossinidia* endosymbiont of *Glossina brevipalpis*	0.224	22.5	*Wigglesworthia* are obligate intracellular bacteria and endosymbionts of the tsetse fly
*Mycoplasma mobile* 163K	0.162	25.0	Fish pathogen
*Mycoplasma mycoides* subsp. *mycoides* SC str. PG1	0.162	24.0	Cattle pathogen
*Borrelia burgdorferi* B31	0.162	28.2	A human pathogen that lives in rodents, and can be transferred to humans via tick bites.
*Borrelia garinii* PBi	0.162	28.1	A human pathogen that lives in rodents, and can be transferred to humans via tick bites.
*Mycoplasma hyopneumoniae* 232	0.156	28.6	Pig pathogen responsible for porcine pneumonia
*Ureaplasma parvum* serovar 3 ATCC 700970	0.155	25.5	Mucosal pathogen of humans
*Mycoplasma hyopneumoniae* 7448	0.154	28.5	Pig pathogen responsible for porcine pneumonia
*Mycoplasma pulmonis* UAB CTIP	0.154	26.6	Mouse pathogen causing murine pneumonia
*Mycoplasma hyopneumoniae* J	0.153	28.5	Pig pathogen causing porcine pneumonia

**Table 3:** The most skewed phage genomes.

Virus type	KLD of amino acid composition from the mean	%GC	Comments on the host
*Spiroplasma kunkelii* virus SkV1_CR2-3x	0.222	22.2	Parasitic lifestyle. Causative agent of Corn Stunt Disease
*Spiroplasma* phage SVTS2	0.211	22.7	Parasitic lifestyle
*Spiroplasma* phage 1-C74	0.199	23.1	Parasitic lifestyle
*Propionibacterium* phage B5	0.192	64.3	A parasite and commensal of humans and other animals that lives in and around sweat glands, sebaceous glands and other areas of the skin (Lood & Collin, 2011)
*Spiroplasma* phage 1-R8A2B	0.186	22.9	Parasitic lifestyle
*Acholeplasma* phage MV-L1	0.183	33.3	N/A^a^
*Mycoplasma* phage phiMFV1	0.167	25.1	Parasitic lifestyle
*Clostridium* phage D-1873 CLG.Contig168	0.153	25.3	N/A^a^
*Mycoplasma* phage P1	0.152	26.8	Parasitic lifestyle
*Clostridium* phage c-st	0.133	26.3	N/A^a^

**Notes.**

a N/A, No metadata available on pathogenesis or lifestyle of the host.

### GC content and amino acid variations among genomes

Amino acid deviation between different phages and bacteria were compared (Figs. 1 and 2). To inspect the functional significance of those differences, we compared the composition of proteins involved in different aspects of metabolism. In this comparison, the null hypothesis was that the compositional bias was randomly distributed among all protein metabolic functional classes, and the alternative hypothesis was that the bias was limited to one or a few functional groups that might contain critically skewed amino acid compositions in some genome. To address this potential source of bias, we used SEED subsystems (Overbeek et al., 2005), collections of genes in pathways or functional associations manually curated by teams of annotators in the SEED database (Aziz, 2010; Aziz et al., 2012). Different subsystems are arranged in a hierarchy of groups.

#### Bacterial genomes

**Figure 3:**
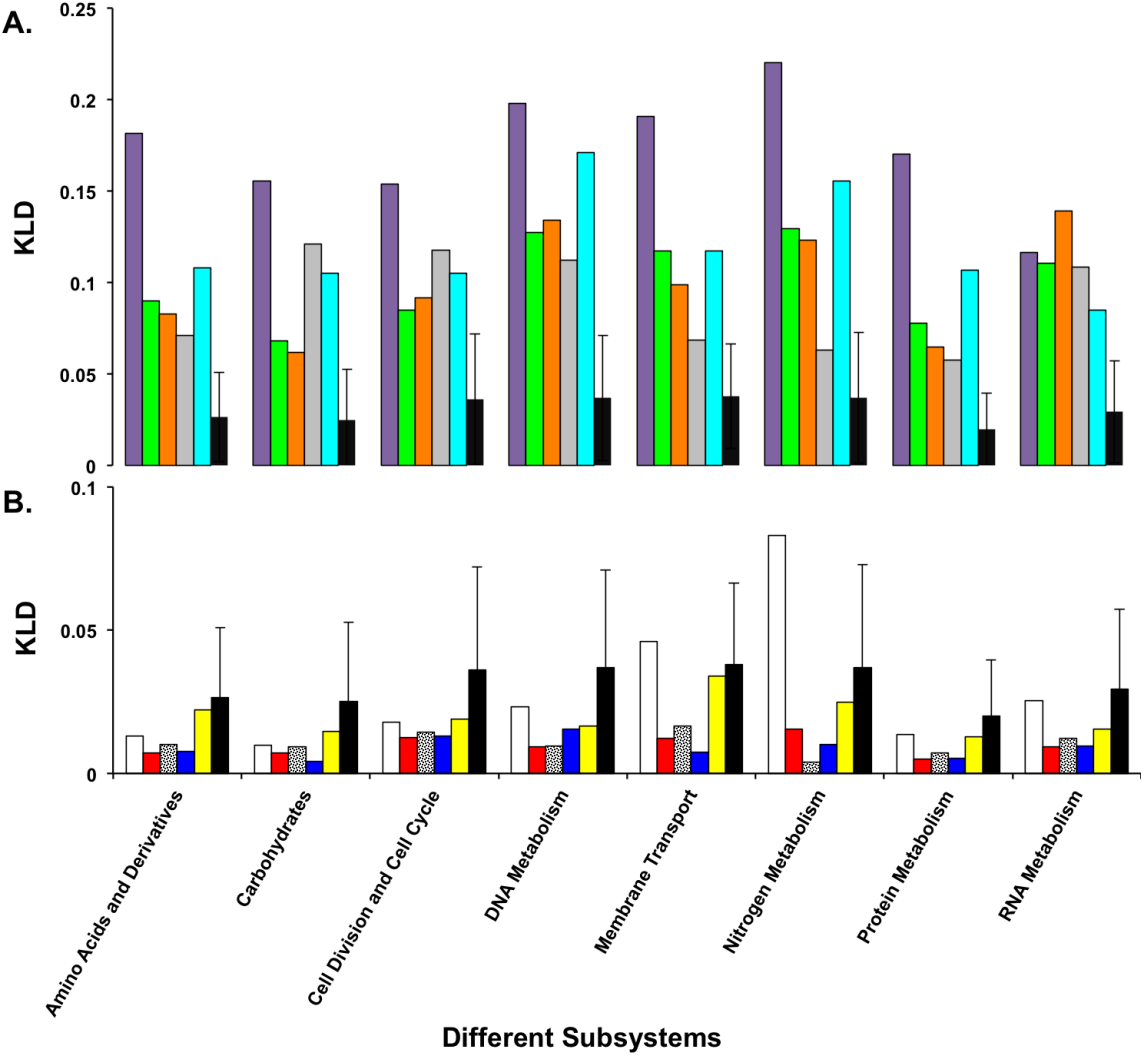
Divergence of amino acid composition and phylogeny. Comparison of the divergence of amino acid composition and phylogenetic group for the most divergent bacterial genomes (A) and the genomes of five bacteria chosen at random (B). In (A) the first five bars are *Wigglesworthia glossinidia, Borrelia garinii, Mycoplasma mycoides, Ureaplasma parvum serovar* and *Buchnera aphidicola* (see Table 2). In (B) the first five bars are *Bifidobacterium adolescentis*, *Bacillus B-14905*, *Nostoc sp. PCC 7120*, *Salmonella bongori 12149*, and *Chlamydophila pneumoniae CWL029*. In both panels, the sixth bar is for the mean of amino acid utilization for each subsystem. (Note the difference in *y*-axis scale between the two panels).

At the time this study was performed there were 31 top-level classifications for protein functions, 229 first–second level classifications (the second level is not unique, but the combination of first and second level is), and 1,078 third level classifications (the subsystems themselves). To investigate whether the amino acid skews in protein composition are dependent on protein function, we calculated KLD for each subsystem’s first level hierarchy in ten bacterial genomes. Five were chosen from the most extremely skewed organisms (Table 2), and five were chosen at random from the remaining genomes. KLD values of the five bacterial species with most skewed amino acid composition greatly differed from the mean for all subsystems, as expected from their overall bias. However, those differences were not restricted to one or a few metabolic process, but were rather consistent across all subsystems tested (Fig. S1), so the hypothesis that the distribution of skewed amino acids is non-random across the genome or that it is dependent of functional categories could not be confirmed. The five control bacterial species, chosen at random, exhibited much less variation in amino acid composition (Fig. 3).

**Figure 4:**
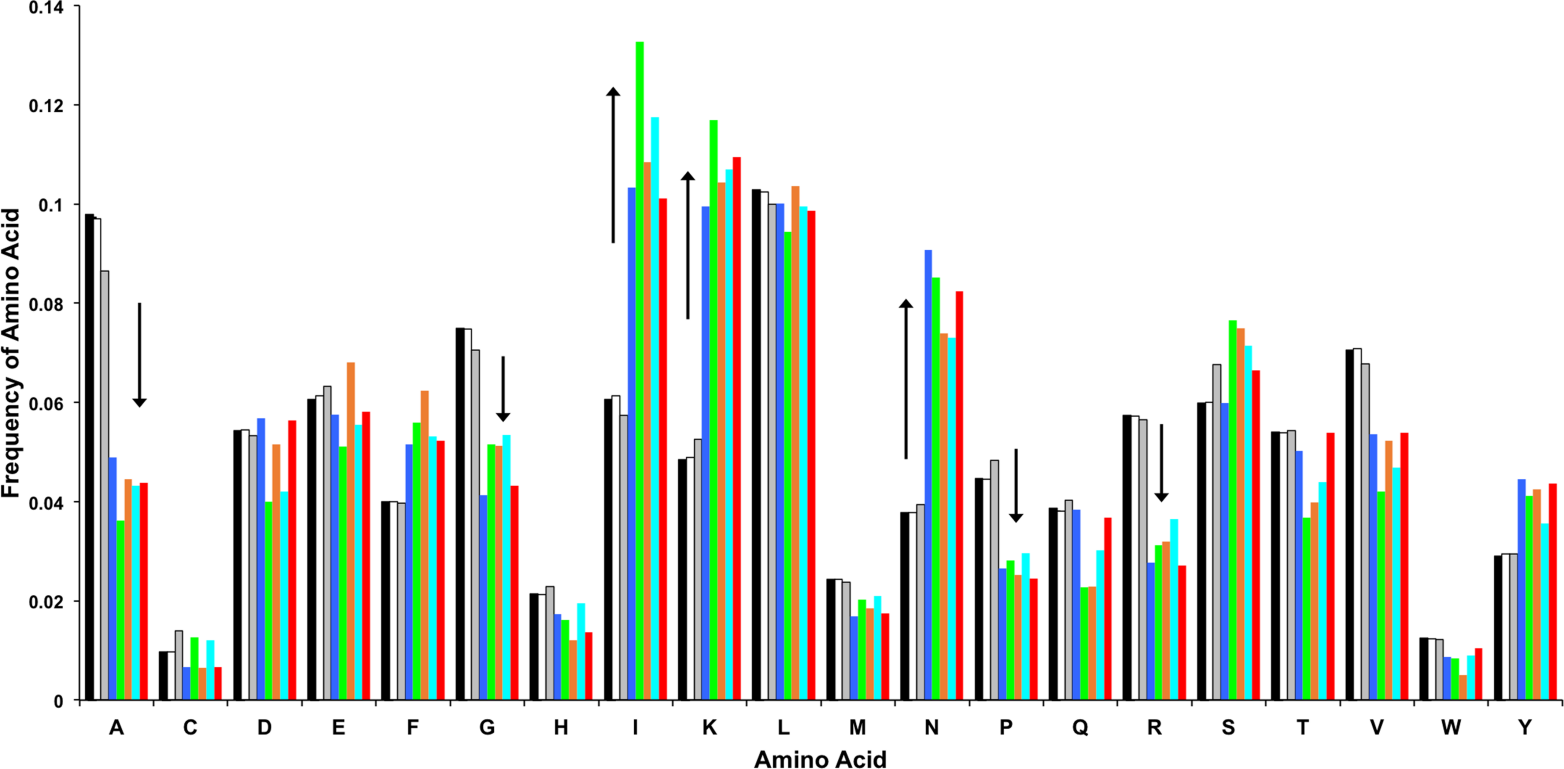
Frequency of each of the twenty amino acids in the three domains of life and the most skewed genomes. The first three bars are the average frequency of amino acid for the three domains Archaea, Bacteria and Eukaryota. The next five bars are for *Ureaplasma parvum serovar, Wigglesworthia glossinidia, Borrelia garinii, Buchnera aphidicola,* and *Mycoplasma mycoides*. Arrows indicate amino acid frequency smaller or larger than the mean for these five bacteria.

To examine whether the compositional skew of bacterial protein sequences was only restricted to one or a few amino acids, we calculated the frequency of occurrence of each amino acid for the five bacterial genomes that have the most skewed amino acid composition (Fig. 4). The null hypothesis was that there would be random changes in the amino acid compositions in these genomes. However, the initial hypothesis was rejected: all five bacterial genomes were found to have significantly reduced their utilization of the amino acids alanine (A), glycine (G), proline (P), and arginine (R), compared to the mean amino acid utilization calculated from all bacteria. This utilization bias appears to have been compensated by an increase in the utilization of the amino acids isoleucine (I), lysine (K) and asparagine (N).

This switch in amino acid utilization has a considerable biological impact because these amino acids are discriminatory in the standard genetic code. A genome consisting entirely of guanosine and cytosine could only encode for alanine (GCC or GCG), glycine (GGC or GGG), proline (CCC or CCG), or arginine (CGC or CGG). In contrast, a genome that contains only adenosine and thymidine could only encode for asparagine (AAT), isoleucine (ATT or ATA), leucine (TTA), lysine (AAA), phenylalanine (TTT), or tyrosine (TAT). Thus, the skew in amino acid composition seems to have been driven by the GC content of the DNA sequence more than the lifestyle, phylogeny, or other characteristics associated with the genome.

**Figure 5:**
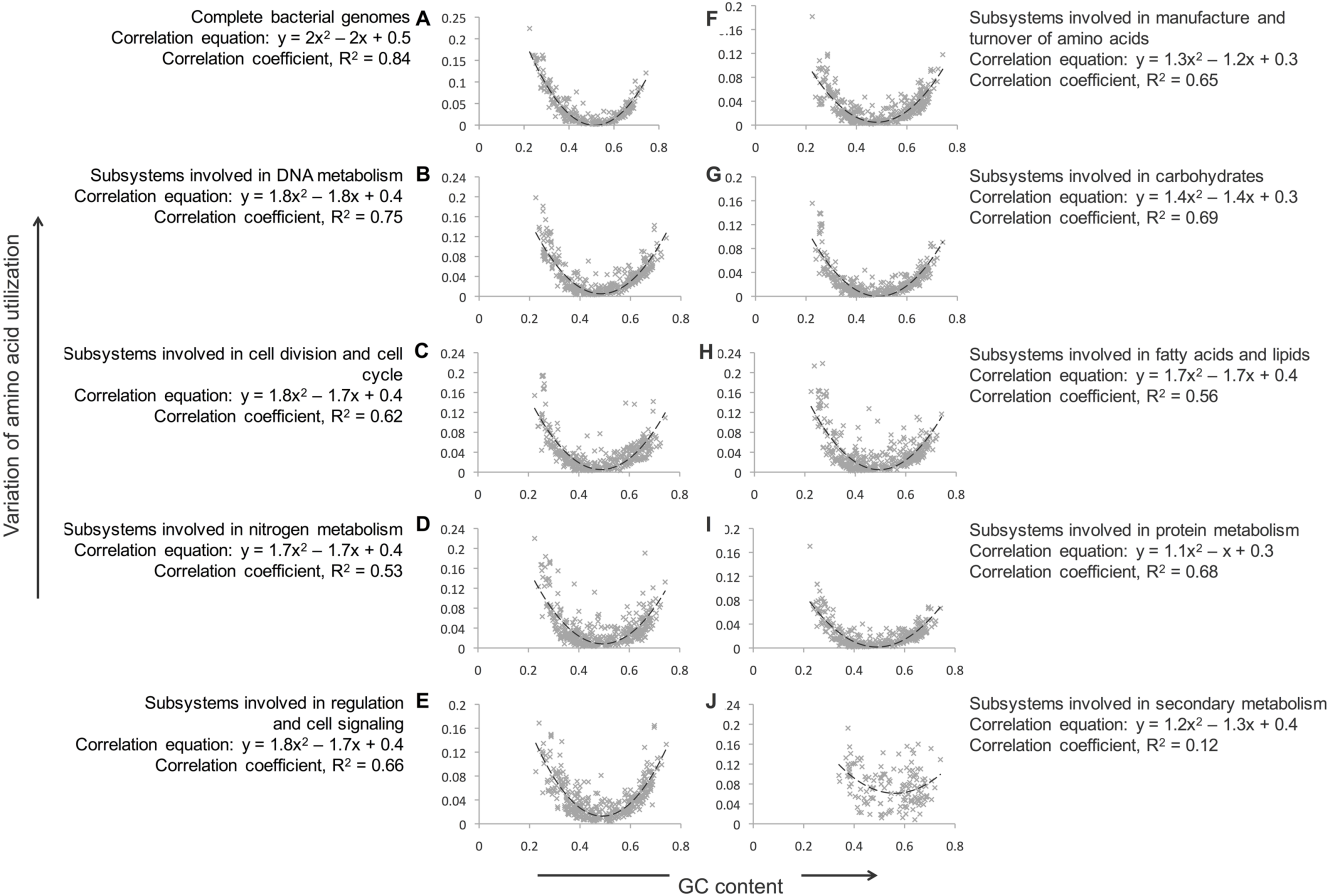
Comparison of KLD and GC-content for all bacterial genomes, and for individual groups of subsystems. The GC content of each genome is plotted on the *x*-axis, and the variation in amino acid composition is shown on the *y*-axis.

The correlation between the percent of sequences that are either guanosine or cytosine (%GC) and the KLD of the amino acid composition to the mean was calculated (Fig. 5). The relationship between %GC and amino acid divergence is given by the equation *y* = 2(*x* − 0.5)^2^, where *x* is the %GC and y is the divergence of amino acid composition (with a square of correlation coefficient, of 0.84). To test whether the correlation is similar for all areas of metabolism, the relationship between %GC and KLD was calculated for the different subsystems shown in Fig. 4. Most subsystems had similar parabolas suggesting that the DNA content and amino acid composition were related. However, the relationship did not hold for the secondary metabolism subsystems (the square of correlation coefficient fell to 0.119, Fig. 5). This suggests that the amino acid profiles of proteins involved in secondary metabolism subsystems are not related to the GC content of the genome. We hypothesize that this may imply that genomic subsystems involved in secondary metabolism are more frequently horizontally transferred than those involved in core metabolism, which are usually highly conserved, and we may be observing the skew of the donor organism rather than the current host.

#### Phage genomes

**Figure 6:**
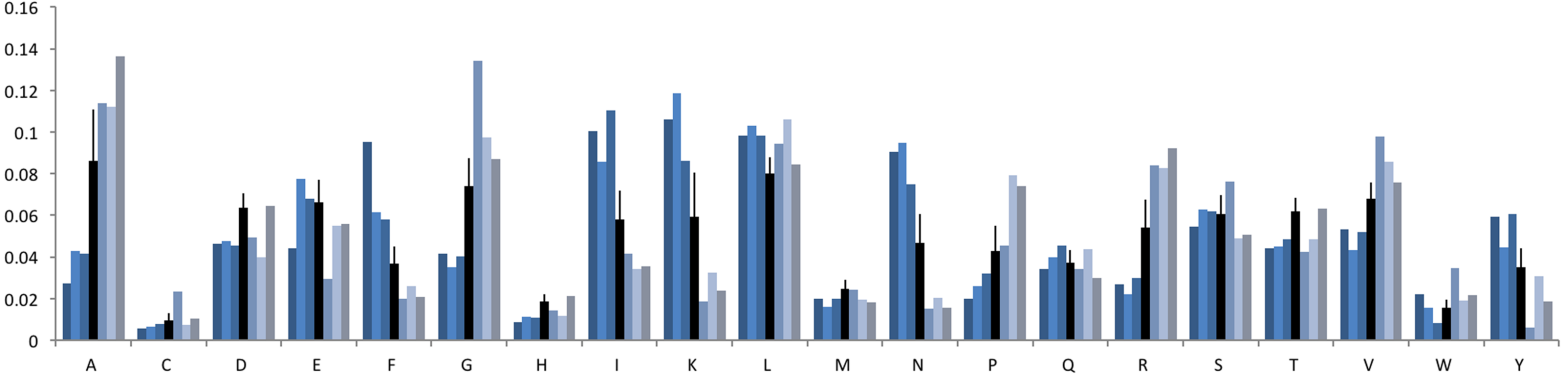
Amino acid frequency in phage genomes. The first three bars are for *Spiroplasma kunkelii* virus SkV1_CR2-3x (GC = 22%), *Mycoplasma* phage phiMFV1 (GC = 25%) and *Sulfolobus islandicus* rod-shaped virus 1 (GC = 25%). These three genomes are GC poor genomes. The fourth bar represents the average frequency of amino acid for 835 phage genomes. The last three bars are for *Propionibacterium* phage B5 (GC = 64%), *Thermus* phage P23-77 (GC = 67%) and *Streptomyces* phage VWB (GC = 71%), which are GC rich genomes.

To examine the amino acid utilization behavior for the most skewed phage genomes, we analyzed three GC-rich and three GC-poor phage genomes in more detail. Similar to bacterial genomes, the amino acid composition also seems to be driven by the GC content for the most skewed phages (Fig. 6). For example, for amino acids lysine (AAA, AAG) and isoleucine (ATT, ATC, ATA), phage genomes with poor GC content have higher frequency but the GC-rich phage genomes have relatively lower frequency compared to the average amino acid utilization among 835 phage genomes.

**Figure 7:**
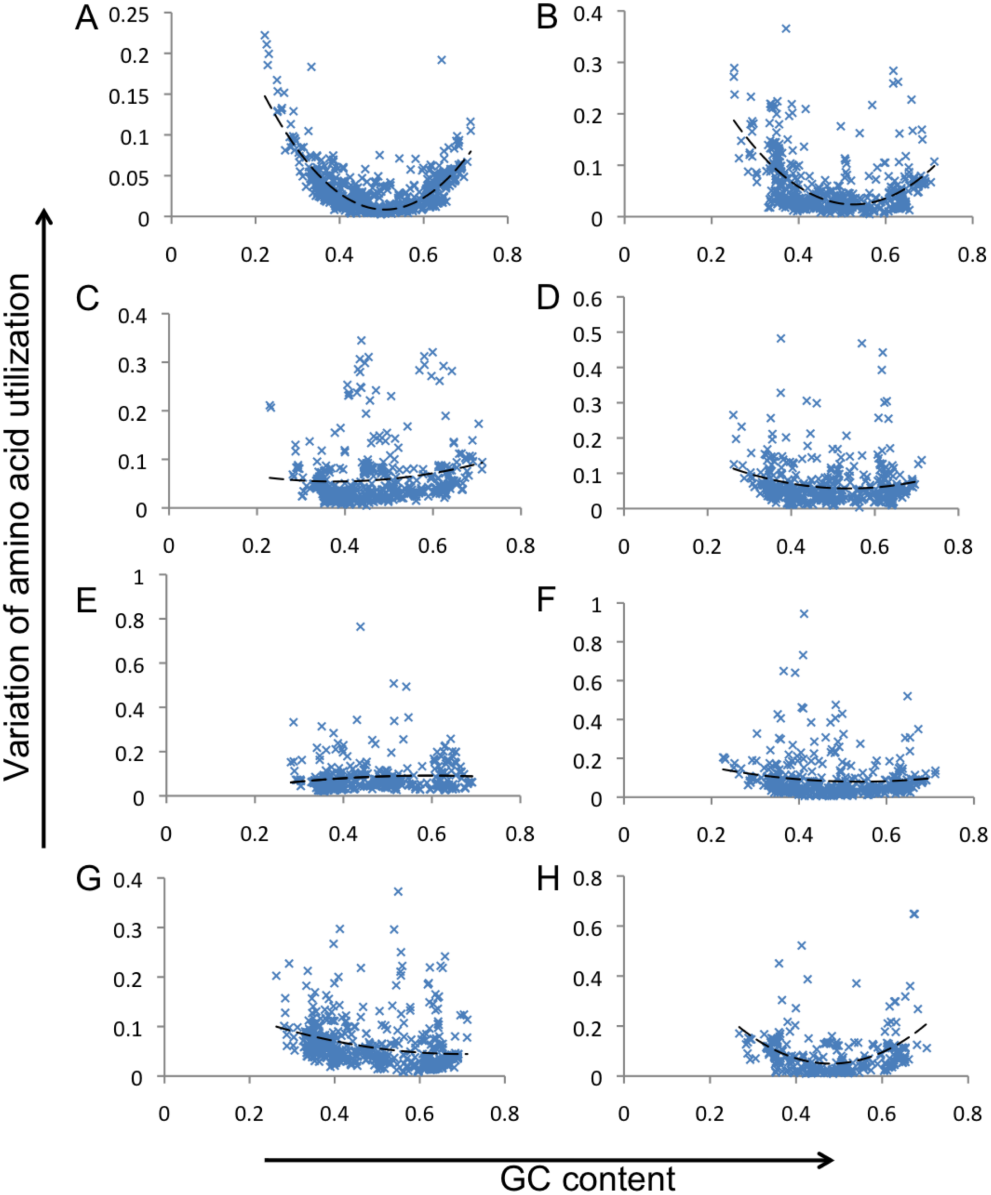
Comparison of KLD and GC-content for all phage genomes. (A), and for individual groups of subsystems (B–H). The GC content of each genome is plotted on the *x*-axis, and the variation in amino acid composition is shown on the *y*-axis. The correlation equation for A–complete phage genomes is *y* = 1.7*x*^2^ − 1.7*x* + 0.44, and the correlation coefficient *R*^2^ = 0.63. In (B), the skews are only shown for those proteins in the phage replication subsystems, and the equation is 2.2*x*^2^ − 2.3*x* + 0.6, with a correlation coefficient = 0.3. In (C), the skews are only shown for those proteins involved in the capsid subsystems, and the representative equation is 0.34*x*^2^ − 0.26*x* + 0.1, with a correlation coefficient = 0.025. In (D), the skews are only shown for those proteins involved in phage head, and the equation is 0.739*x*^2^ − 0.79*x* + 0.27, with a correlation coefficient = 0.029. In (E), the skews are only shown for proteins in subsystems involved in phage lysis, and the equation is −0.31*x*^2^ + 0.37*x* − 0.189, with a correlation coefficient = 0.01. In (F), the skews are only shown for those proteins in experimental subsystems (mostly uncharacterized proteins), and the equation is 0.65*x*^2^ − 0.7*x* + 0.26, with a correlation coefficient = 0.01. In (G), the skews are only shown for those proteins involved in phage tail subsystems, and the equation is 0.285*x*^2^ − 0.4*x* + 0.1856, with a correlation coefficient = 0.095. In (H), the skews are only shown for those proteins in phage tail fiber subsystems, and the equation is 3.2*x*^2^ − 3.09*x* + 0.79, with a correlation coefficient = 0.15.

Like with bacterial genomes, deviation of the amino acid composition (KLD) in phage genomes strongly correlates with their GC% (Fig. 7A). The relationship is *y* = 1.7(*x* − 0.5)^2^, where *x* is the %GC and *y* is the KLD (with a square of correlation coefficient of 0.84). The relationship between KLD and GC content is statistically different for bacteria and phages (*p*-value < 10^6^, details in Supplemental Information). To check whether the variation of amino acid utilization is restricted to one or a few subsystems, KLD was calculated for several phage subsystems in all phage genomes. No strong correlation was observed between functional category and GC%, with the exception of the phage replication subsystem (correlation coefficient, *R*^2^ = 0.3). This lack of correlation can be explained by the highly diverse nature of phages, which have different mutational and gene transfer dynamics than bacteria.

### GC content and amino acid frequency within genomes

As the variation of amino acid in a genome (for both bacteria and phages) has a strong correlation with the genome’s GC content, the frequency of amino acid utilization was calculated and plotted against %GC for 446 bacterial genomes and 835 phage genomes (Figs. S2, S3). The correlation between each amino acid and %GC for both phages and bacterial genome follows a similar pattern, although, for phages, there is almost no correlation between the amino acid deviation and %GC for most subsystems.

## Discussion

A complete genome, unlike random sequences, represents an evolutionary successful set of nucleotides, whose combination encodes a functioning organism that survived selection pressure through the evolutionary times and that is still evolvable (Hogeweg, 2012). The accrual of complete genome sequences provides an invaluable resource for exploring the different means by which the combination of four nucleotides (A, G, C, and T/U) encodes life forms able to survive the different environments of our planet. Because the genetic code is relatively simple yet redundant, studying the information content inherent to complete genome sequences is expected to enable the discovery of various properties of a genome’s architecture (e.g., gene order and density, and genome symmetry), compositional bias (e.g., GC content and skews), coding potential (i.e., all possible amino acid combinations it can encode), codon usage preferences, and epistatic parameters. Such properties can be correlated with functional aspects encoded by the genome and can shed the light on its natural history, allowing the study of the organism’s evolution (Adami, 2012; Gautier, 2000; Nasrallah & Huelsenbeck, 2013). For example, epistatic parameters and their statistical analysis gave clues on the evolution of influenza A virus (Nshogozabahizi, Dench & Aris-Brosou, 2017).

Additionally, these compositional and informational properties can be exploited to develop better strategies of genome interpretation. For example, the information theory, compositional statistics, and genome topography have been extensively used in gene prediction, genome assembly, RNA finding (Bernhart & Hofacker, 2009; Li et al., 2010), and the prediction of horizontal gene transfer (Davis & Olsen, 2010; Langille & Brinkman, 2009; Mrazek & Karlin, 1999; Ochman, Lawrence & Groisman, 2000; Price, Dehal & Arkin, 2008). Lately, more sophisticated analyses aimed at differentiating between informative and less informative sequences in viral genome analyses and metaviromics (Watkins & Putonti, 2017).

In this study, we explored the possibility of exploiting the coding potential and amino acid distribution biases within complete bacterial and phage genomes for better interpreting sequence fragments (e.g., metagenomic reads), and predicting which sequence reads within large data sets are likely to encode proteins. To this end, we calculated KLD, a measure of information divergence, for a set of bacterial and phage genomes, and compared the distribution of amino acids in different protein-coding sequences in an attempt to use this metric as a measure of how much those sequences deviate from the standard—the standard being defined by the combined amino acid distribution in all genomes.

We found a significant difference in amino acid utilization between phylogenetic groups of bacteria and phages. In addition, we found an enrichment of intracellular endosymbiotic or pathogenic bacterial genomes among those with the most skewed amino acid utilization profiles, or an enrichment of phages that infect such bacteria. Whereas amino acid skews did not seem to be restricted to a particular functional subsystem, they strongly correlated with the GC content of bacterial genomes (Salzberg et al., 1998; Kelley et al., 2012).

Many studies have shown that the GC content of a genome influences the frequencies of oligonucleotides and thus amino acid composition of its encoded proteome, which reflect the lifestyle of the organism (e.g., Bharanidharan et al., 2004; Bohlin, Skjerve & Ussery, 2008; Lobry, 1997; Najafabadi & Goodarzi, 2004; Rocha & Danchin, 2002; Ren et al., 2017). It is also correlated with the GC proportion of all the synonymous codons for a particular amino acid and has an impact on codon/amino acid usage (Davis & Olsen, 2010; Gerdol et al., 2015). In this work, we demonstrate how the GC content is driving the divergence of amino acid composition in bacterial genomes away from the mean composition through the use of KLD divergence. All five bacterial genomes with the highest amino acid compositional skew have low GC content (ranging from 22% to 28%), and consequently fewer alanine, glycine, proline and arginine residues in their encoded proteins. Their relative inability to encode these amino acids, and their substitution of them with isoleucine, lysine, and asparagine explains the significant skews seen in the protein sequences (Fig. 3).

Conversely, GC-rich bacteria have fewer codons for phenylalanine, isoleucine, lysine, asparagine, and tyrosine, but compensate with alanine, glycine, proline, and arginine. Therefore, both GC-rich and GC-poor bacteria have the most divergent amino acid compositions, while bacteria with an average GC content have an average amino acid composition (Fig. 4). The correlation coefficient (*R*^2^ = 0.84) suggests a strong relationship between GC% and KLD. However, as the relationship is not linear, we propose that this relationship gives a better understanding of the correlation between GC content and the variation of amino acid utilization.

The divergence of amino acid composition is not restricted to one or a few functional categories, but is common across all subsystems. For almost all subsystems involved in primary metabolism, the relationship closely follows similar quadratic equations with high correlation coefficients. In contrast, subsystems involved in secondary metabolism appear to have a poor correlation between GC content and amino acid composition. Two possible reasons for this are a high level of horizontal gene transfer in genes within these subsystems, ameliorating the amino acid utilization, or the poorer quality of annotation of secondary metabolism in diverse organisms. Only 167 bacteria have an annotated secondary metabolic subsystem, and most of those have GC content between 40% and 60%.

Some differences were noted in the trends of KLD variation between bacterial and phage genomes. Phages have slightly lower KLD than bacterial genomes, albeit not strongly statistically significant, which suggests that bacterial genomes may have more homogeneous amino acid frequencies than phage genomes. This could be because bacterial genomes are more conserved than those of phages, which could be the result of strong negative selection pressure exerted on core metabolic and information transfer subsystems in bacteria, as opposed to the lack of core sets of genes among known phages. Moreover, phage population dynamics, their mode of replication, and their rapid turn over result in highly variable, mosaic genomes.

It is worth noting that many of the phages with most skewed amino acid composition infect bacterial endosymbionts or obligate parasites. This observation is consistent with our hypothesis that KLD values reflect genome conservation, a phenomenon exaggerated in genomes with limited environment and poor exchange with other sources of DNA. Endosymbionts and intracellular parasites are confined to closed environments, and thus their genomes have the highest variation from the average amino acid distribution. On the other hand, genomes of bacteria (or phages that infect them) living in open environments, which are continuously changing have less variation from the average distribution.

Additional evidence for this correlation between KLD and sequence conservation comes from the observation that only within the “phage replication” subsystem, the correlation between KLD and GC content is strong: phage replication genes are among the very few genes that are conserved across most phage types.

Interestingly, *Mycoplasma* species, which are known to be intracellular parasites, were the only bacterial species with the most skewed KLD values (Table 2) and which host phage genomes with the highest KLD skew as well (Table 3). In a recent study, where the KLD was calculated for tetranucleotides in bacterial genomes (Bohlin et al., 2012), *Mycoplasma* sp. was also considered as the most skewed bacterial genome.

##  Supplemental Information

10.7717/peerj.4026/supp-1 Supplemental Information 1 Supplemental material

10.7717/peerj.4026/supp-2 Supplemental Information 2 Raw data file: KLD calculations for bacterial genomes

10.7717/peerj.4026/supp-3 Supplemental Information 3 Raw data file: KLD calculations for phage genomes

10.7717/peerj.4026/supp-4 Supplemental Information 4 Raw data file: amino acid frequency calculations

## Additional Information and Declarations

### Data Availability

The following information was supplied regarding data availability: The raw data has been provided in Supplemental Files.
