# Supplementary material for: Kullback Leibler divergence in complete bacterial and phage genomes
Source: PeerJ. 2017 Nov 30;5:e4026. doi: 10.7717/peerj.4026 (PMC5712468; doi:10.7717/peerj.4026)

Kullback Leibler Divergence in Complete Bacterial and Phage Genomes

Sajia Akhter, Ramy K. Aziz, Mona T. Kashef, Eslam S. Ibrahim, Barbara Bailey, Robert A. Edwards

Supplemental Material

T-test for testing the relationship between GC content and amino acid variations for bacteria and phage samples:

There are two samples: bacteria and phages.
The sample size of bacteria, m = 372
The linear model of bacterial sample: $y = 2x^2 - 2x + 0.5$ … (1)
The sample size of phages, n = 835
The linear model of phage sample: $y = 1.7x^2 – 1.7x + 0.44$ … (2)

The general form of these equations is: $y = \beta_0 + \beta_1 x + \beta_2 x^2$
We want to test whether the coefficients (β_1 and β_2) of these two equations are significantly different:

H_0: $\beta_1^1 = \beta_1^2$ and $\beta_2^1 = \beta_2^2$
H_A: $\beta_1^1 \neq \beta_1^2$ and $\beta_2^1 \neq \beta_2^2$
where β_1^1 is the β_1 coefficient of equation 1 (bacterial sample) and β_1^2 is the β_1 coefficient of equation 2 (phage sample).

T test for two independent unequal sample sizes:

$$t = \frac{\bar{\beta}_p - \bar{\beta}_b}{\sqrt{SE(\beta_p)^2 + SE(\beta_b)^2}}$$, where SE is the standard error.

Here, degree of freedom, df = m-2 + n -2 = 372 -3 + 835 -3 = 1201

For coefficient β_1, p value is 1.058481e-07, and for coefficient β_2, p value is 1.631291e-06. So we can reject the null hypothesis.

This means Equation 1 and Equation 2 are significantly different.

Supplemental Figure 1: Comparison of the divergence of amino acid composition and the phylogenetic group for the most divergent bacterial genomes. The first five bars represent *Wigglesworthia glossinidia*, *Borrelia garinii*, *Mycoplasma mycoides*, *Ureaplasma parvum* serovar and *Buchnera aphidicola*. The sixth bar is for the mean of amino acid utilization for each subsystem.

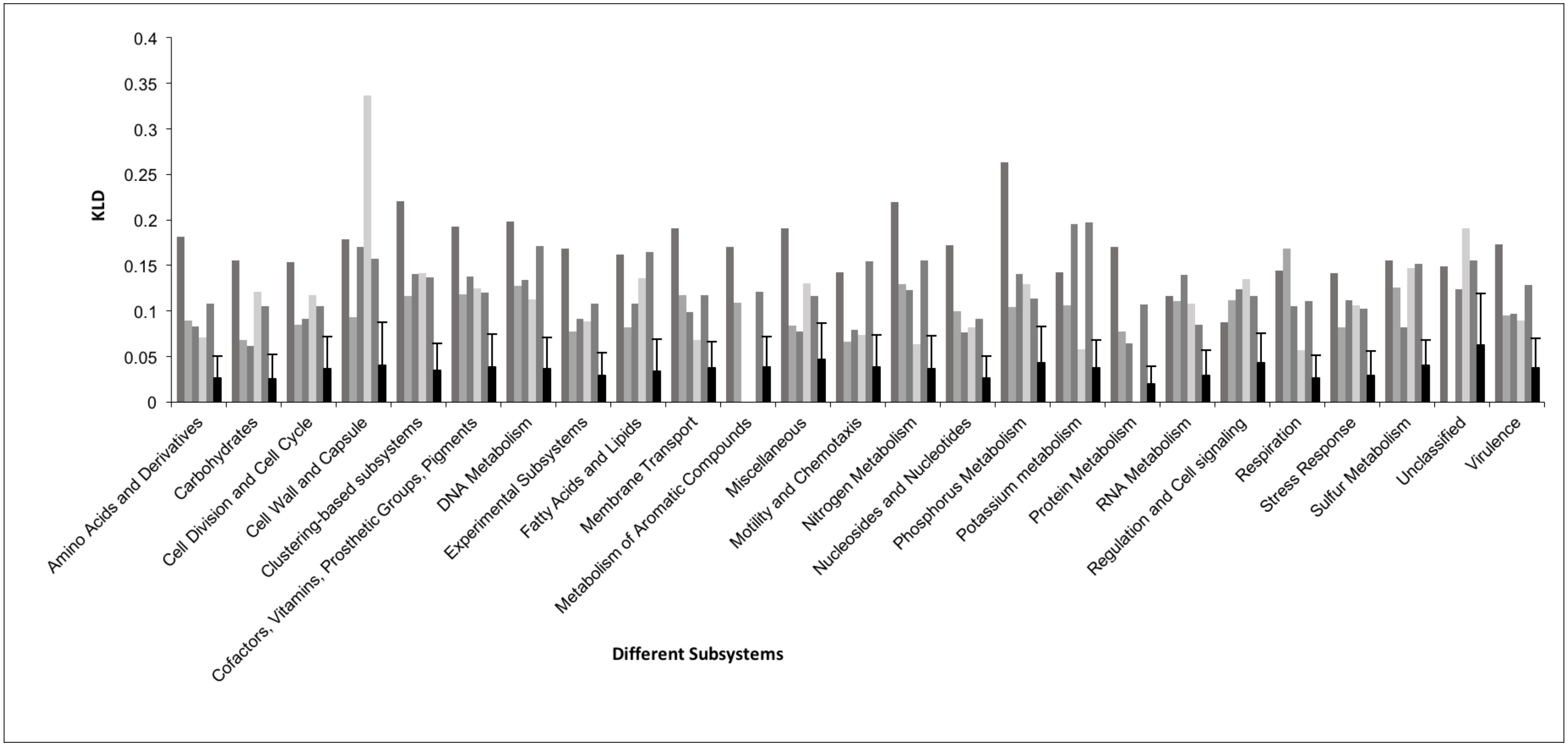

Supplemental Figure 2: Amino acid frequency of 446 bacterial genomes vs. GC%

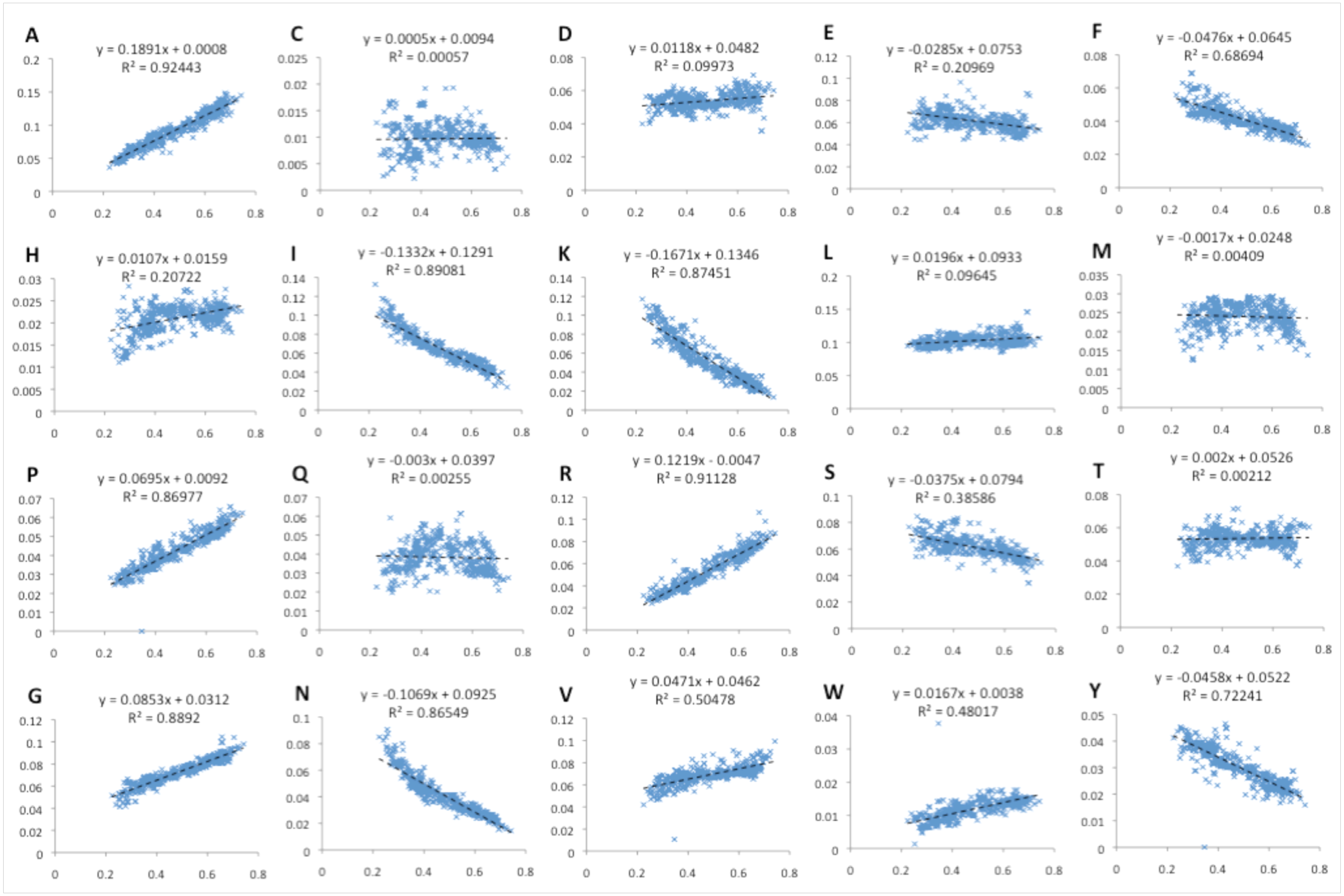

Supplemental Figure 3: Amino acid frequency of 835 complete phage genomes vs. GC%

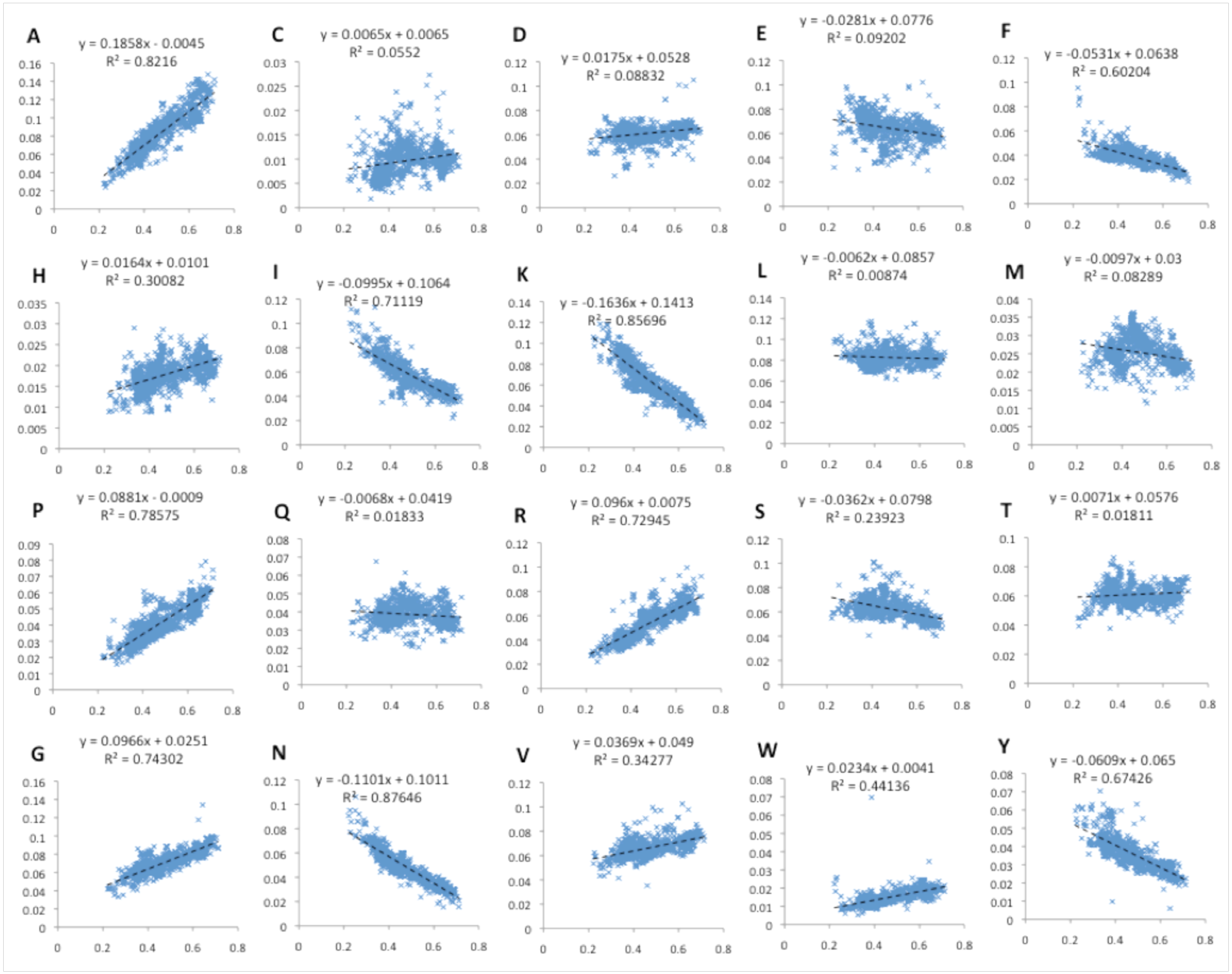

Supplement: Supplemental Information 1 [file peerj-05-4026-s001.pdf]
